# Matching-adjusted indirect comparison of tislelizumab plus lenvatinib versus sintilimab plus bevacizumab biosimilar as first-line treatment for unresectable hepatocellular carcinoma

**DOI:** 10.3389/fimmu.2025.1594935

**Published:** 2025-06-23

**Authors:** Kunyuan Wang, Chang Liu, Xiaoling Song, Na Zhao, Xin Zheng

**Affiliations:** ^1^ Department of Infectious Diseases and Hepatology, State Key Laboratory of Organ Failure Research, Nanfang Hospital, Southern Medical University, Guangzhou, China; ^2^ Guangdong Provincial Key Laboratory of Viral Hepatitis Research, Nanfang Hospital, Southern Medical University, Guangzhou, China; ^3^ Division of Abdominal Tumor Multimodality Treatment, Cancer Center, West China Hospital, Sichuan University, Chengdu, China; ^4^ Department of General Surgery and Laboratory of General Surgery, Shanghai Key Laboratory of Biliary Tract Disease Research, Xinhua Hospital, Shanghai Jiao Tong University School of Medicine, Shanghai, China; ^5^ Global Statistics and Data Science, BeiGene (Shanghai) Co., Ltd., Shanghai, China; ^6^ Department of Hepatobiliary Surgery, The First Affiliated Hospital of Xi’an Jiaotong University, Xi’an, China

**Keywords:** hepatocellular carcinoma, matching-adjusted indirect comparison, tislelizumab, lenvatinib, sintilimab, bevacizumab

## Abstract

**Background:**

Programmed cell death-1/programmed cell death-ligand 1 (PD-[L]1) inhibitors plus bevacizumab (or biosimilars) or tyrosine kinase inhibitors (TKIs) have been widely used for the first-line treatment of patients with unresectable hepatocellular carcinoma (uHCC). However, no head-to-head trials have compared the efficacy outcomes between these two combination regimens. Therefore, an unanchored matching-adjusted indirect comparison (MAIC) was conducted to evaluate the comparative efficacy of tislelizumab plus lenvatinib versus sintilimab plus bevacizumab biosimilar.

**Methods:**

Individual patients from the BGB-A317-211 study (NCT 04401800) for tislelizumab plus lenvatinib were adjusted to match the population from the ORIENT-32 (NCT 03794440) for sintilimab plus bevacizumab biosimilar through an unanchored MAIC. Odds Ratios (ORs) of objective response rates (ORR) and disease control rates (DCR), and hazard ratios (HRs) of progression-free survival (PFS) and overall survival (OS) were evaluated to quantify the relative treatment effect between the two treatment regimens after population matching. Sensitivity analyses were performed by sequentially removing one variable in the matching and adjusting the population through simulated treatment comparison (STC).

**Results:**

After matching, baseline characteristics were balanced between the tislelizumab plus lenvatinib group (effective sample size [ESS] = 49, ESS/N = 79.03%) and sintilimab plus bevacizumab biosimilar group (N = 380). MAIC analysis indicated that tislelizumab plus lenvatinib group showed significantly higher ORR per RECIST v1.1 (OR = 2.56, 95% CI 1.40-4.63; *p* = 0.0027), higher DCR (OR = 3.81, 95% CI 1.62-11.20; *p* = 0.0013), longer PFS (HR = 0.56, 95% CI 0.37-0.84, *p* = 0.0054), and improved OS (HR = 0.43, 95% CI 0.25-0.74, *p* = 0.0023), compared to sintilimab plus bevacizumab biosimilar group. Sensitivity analysis by two different methods supported the findings from the primary MAIC analysis.

**Conclusions:**

This MAIC analysis demonstrated that tislelizumab plus lenvatinib achieved superior efficacy, with higher ORR and longer PFS and OS compared to sintilimab plus bevacizumab biosimilar in untreated Chinese patients with uHCC.

## 1 Introduction

Hepatocellular carcinoma (HCC) comprises approximately 75%-85% of primary liver cancer cases and is the fourth-leading cause of cancer-related death worldwide (1, 2). Owing to the late presentation of symptoms, more than 70%-80% of HCC patients are diagnosed at advanced stages and are not eligible for hepatic resection (3). Programmed cell death-1/programmed cell death-ligand 1 (PD-[L]1) inhibitors and anti-vascular endothelial growth factor (VEGF) or tyrosine kinase inhibitor (TKI) combination treatments have been widely applied in the first-line treatment of patients with unresectable HCC (uHCC) (4–6). In China, the first-line recommended immunotherapy combination strategies included both PD-(L)1 plus anti-VEGF or plus TKIs, for instance, atezolizumab plus bevacizumab, sintilimab plus bevacizumab biosimilar, and camrelizumab plus apatinib. Another immunotherapy combination that has gained attention is lenvatinib (a multi-targeted TKI) plus a PD-1 inhibitor. Although in the LEAP-002 study, lenvatinib plus pembrolizumab failed to meet its dual primary endpoints of overall survival (OS) and progression-free survival (PFS) in the global intent-to-treatment (ITT) population, recent Asian subgroup analysis revealed meaningful OS (median, 26.3 vs. 22.4 months; hazard ratio [HR] = 0.73; 95% confidence interval [CI], 0.55-0.96) and PFS (median, 8.3 vs. 6.5 months; HR = 0.71; 95% CI, 0.56-0.91) improvement trend over lenvatinib (7, 8). This suggests that lenvatinib plus a PD-1 inhibitor is a promising first-line treatment strategy for uHCC patients in Asian populations.

Tislelizumab is a PD-1 inhibitor designed to minimize binding to Fcγ receptors on macrophages to limit antibody-dependent cellular phagocytosis, a potential mechanism contributing to anti-PD-1 therapy resistance (9). Tislelizumab demonstrated a non-inferior OS benefit compared to sorafenib (median, 15.9 vs. 14.1 months; HR = 0.85; 95% CI, 0.71-1.02) in a global randomized phase III study (RATIONALE-301) as a first-line treatment option for patients with uHCC (10). Meanwhile, in a prospective, multicenter, phase II trial (BGB-A317-211 study), we explored the efficacy and safety of tislelizumab plus lenvatinib in treatment-naïve patients with uHCC. This study demonstrated the promising clinical efficacy of this combination approach with objective response rates (ORR) of 38.7% and a 12-month OS rate of 88.6% (11). In China, the combination therapy of sintilimab and a bevacizumab biosimilar received regulatory approval and has since been incorporated into multiple indirect comparisons (12, 13). Considering that both tislelizumab plus lenvatinib and sintilimab plus bevacizumab biosimilar are broadly used treatment regimens in clinical practice in China, we attempted to compare the efficacy outcomes between these two regimens.In the absence of direct comparisons with head-to-head clinical trials, an indirect treatment comparison (ITC) can be conducted. Matching-adjusted indirect comparison (MAIC) is a method to reduce bias in indirect treatment comparisons by aligning individual patient-level data (IPD) from one trial with the aggregate data reported for the comparator trial (14). By weighting IPD to match the characteristics of the comparator trial population, MAIC enables the estimation of relative efficacy across balanced trial populations (15). MAIC is a recommended methodology for indirect comparisons used routinely in submissions to health authorities like the National Institute for Health and Care Excellence (NICE) in the United Kingdom, which can provide comparative effectiveness evidence among studies and inform clinical decision-making (14). Several studies have reported the application of MAIC in the context of HCC, demonstrating its utility in evaluating the relative benefits of different treatment strategies when direct comparisons are lacking.

In the present study, we conducted an MAIC analysis to compare the relative efficacy between the treatment regimens of tislelizumab plus lenvatinib versus sintilimab plus bevacizumab biosimilar in Chinese patients with uHCC. Given that the BGB-A317-211 study is a single-arm trial, an unanchored MAIC analysis was used. Population adjustment through simulated treatment comparison (STC), as well as a leave-one-out analysis, was also conducted to assess the robustness of the MAIC results.

## 2 Methods

### 2.1 Data sources

An unanchored MAIC was conducted using IPD from patients treated with tislelizumab plus lenvatinib in the BGB-A317-211 (N = 62) trial and published aggregate data from the ORIENT-32 trial (sintilimab plus bevacizumab biosimilar, N = 380) (11, 16). The analysis followed the NICE Decision Support Unit (DSU) guidelines for population-adjusted indirect comparisons (17). No ethics committee review board was required for this study as it was based on a *post hoc* analysis of previously published data.

The characteristics of the BGB-A317-211 (NCT04401800) and ORIENT-32 (NCT03794440) have been thoroughly described in the literature (11, 16). BGB-A317-211 (median follow-up time of 15.7 months with a data cutoff date of December 1, 2022) was a single-arm, open-label, multicenter phase II study of tislelizumab plus lenvatinib as the first-line treatment in Chinese patients with uHCC. ORIENT-32 (median follow-up time of 15.8 months as of August 15, 2020) was a randomized, open-label, phase II-III study conducted in systemic treatment naïve Chinese uHCC patients; the phase II portion in ORIENT-32 served as a single-arm safety run-in with patients receiving the sintilimab plus bevacizumab biosimilar combination, and the phase III portion was a randomized, controlled trial, in which patients were randomly assigned to receive either sintilimab plus bevacizumab biosimilar or sorafenib treatment.

### 2.2 Study comparisons

A compatibility assessment was performed through a comparative review of the study design, study population, inclusion and exclusion criteria, outcome definitions, and baseline characteristics of the trial populations to assess the similarities and differences between the two trials. Differences that could potentially impact the results were adjusted in the analyses where possible.

The two trials, both conducted in multiple centers in China, exhibited similarities in key eligibility criteria, tumor assessment criteria and frequency, and definitions of efficacy endpoints, showing sufficient inter-study similarities to allow for comparison (**Table 1**). However, imbalances in baseline characteristics between studies necessitated a population adjustment method to reduce bias when comparing tislelizumab plus lenvatinib to sintilimab plus bevacizumab biosimilar (**Table 2**).

**Table 1:** Summary of study designs of the BGB-A317-211 and ORIENT-32 trials.

Characteristics	BGB-A317-211	ORIENT-32
Clinical trial identifier	NCT 04401800	NCT 03794440
Study type	Phase II, single-arm multicenter, open-label	Phase II-III, randomized, multicenter, open-label
Data cut-off	February 18, 2024	August 15, 2020
Median follow-up	15.7 months (range, 0.9-27.4)	15.8 months (IQR, 15·2-16·1)
Patient selection and key inclusion criteria	Histologically or cytologically confirmed unresectable locally advanced or metastatic HCC	Histologically or cytologically diagnosed or clinically confirmed unresectable or metastatic HCC
	Aged 18-70 years	Aged ≥18 years
	BCLC stage C or B disease	BCLC stage C or B disease
	ECOG PS ≤1	ECOG PS ≤1
	Had no prior systemic therapy	Had received no previous systemic therapy for advanced or metastatic disease
	Child-Pugh liver function score of 7 or less	Child-Pugh liver function score of 7 or less
Regimen and dosage	TIS (200 mg on day 1) plus LEN (12 mg [body weight ≥ 60 kg] or 8 mg [body weight < 60 kg] orally taken once daily), every 3 weeks	Treatment arm in phase III part: SIN plus (200 mg) BEV biosimilar (15 mg/kg body weight), every 3 weeks
Primary efficacy endpoints	IRC-assessed ORR per RECIST v1.1	OS, and IRRC-assessed PFS per RECIST v1.1
Key secondary efficacy endpoints	IRC-assessed ORR per mRECIST, DCR and PFS per RECIST and mRECIST, and OS	ORR and DCR per RECIST and mRECIST, and PFS per mRECIST
Definition of outcome	ORR, the proportion of patients with CR or PR as their best overall response; DCR, the proportion of patients with the best overall response of CR, PR, or SD; PFS, time from the first dose of study medication to PD or death; OS, the time from the first dose of study medication to death.	ORR, the percentage of patients whose best overall response was CR or PR; DCR, the proportion of patients who had a CR, PR, or SD; PFS, the time from the first dose of the study drug to the first documented PD or death from any cause; OS, the time from the first dose of the study drug to death from any cause.
Tumor assessment criteria	RECIST v1.1, mRECIST, and iRECIST	RECIST v1.1 and mRECIST
Tumor assessment schedule	Every 6 weeks in the first year of treatment, and every 9 weeks thereafter	Every 6 weeks until week 48, and then every 12 weeks.

IQR, interquartile range; BCLC, Barcelona Clinic Liver Cancer; ECOG PS, Eastern Cooperative Oncology Group Performance Status; TIS, tislelizumab; LEN, Lenvatinib; SIN, sintilimab; BEV, bevacizumab; IRC, independent review committee; IRRC, independent radiological review committee; RECIST v1.1, Response Evaluation Criteria in Solid Tumors version 1.1; mRECIST, modified RECIST; iRECIST, immune Response Evaluation Criteria in Solid Tumors; DCR, disease control rate; ORR, objective response rate; CR, complete response; PR, partial response; SD, stable disease; PD, disease progression.

**Table 2:** Baseline characteristics of BGB-A317-211 versus ORIENT-32 before and after matching.

Characteristics, %	BGB-A317-211		ORIENT-32^*^
	Before MAIC (naïve)	After MAIC	N = 380
	N = 62	ESS = 49	
Age ≥ 53	50.0	50.0	50.0
Male sex	82.3	87.9	87.9
ECOG PS = 0	62.9	48.2	48.2
BCLC stage B	25.8	14.7	14.7
AFP ≥ 400 ng/mL	41.9	43.4	43.4
Macrovascular invasion and/or extrahepatic metastasis	62.9	79.7	79.7

^*^Sintilimab plus bevacizumab biosimilar group of ORIENT-32 trial. ESS, effective sample size; AFP, alpha-fetoprotein; BCLC, Barcelona Clinic Liver Cancer; ECOG PS, Eastern Cooperative Oncology Group Performance Status; SIN, sintilimab; BEV, bevacizumab.

Summaries and analyses of endpoints were based on the patients from the BGB-A317-211 study and the sintilimab plus bevacizumab biosimilar group of the phase III part of the ORIENT-32 study. Both trials reported treatment response (ORR and disease control rate [DCR]) assessed by an independent review committee per response evaluation criteria in solid tumors (RECIST) v1.1 and modified RECIST (mRECIST) criteria, PFS per RECIST v1.1, and OS. In the BGB-A317-211 study, the efficacy outcomes of tislelizumab plus lenvatinib were assessed based on the efficacy evaluable analysis set, which included all patients who had measurable disease at baseline (per RECIST v1.1) and at least one evaluable post-baseline tumor assessment unless treatment was discontinued for disease progression or death before the first assessment. In the ORIENT-32 study, PFS and OS in the sintilimab plus bevacizumab biosimilar group were analyzed based on the ITT population, and treatment response (ORR and DCR) was assessed based on the response-evaluable population who had at least one tumor assessment or died before the first scheduled tumor assessment. Tumor assessment was performed every 6 weeks in the first year of treatment in both studies, every 9 weeks in the BGB-A317-211 study, and every 12 weeks in ORIENT-32 study thereafter. We selected the following efficacy outcomes for comparison between these two trials: independent review committee (IRC)/independent radiological review committee (IRRC)-assessed ORR and DCR per RECIST v1.1 and mRECIST, IRC/IRRC-assessed PFS per RECIST v1.1, and OS.

### 2.3 Statistical analysis

**Figure 1:**
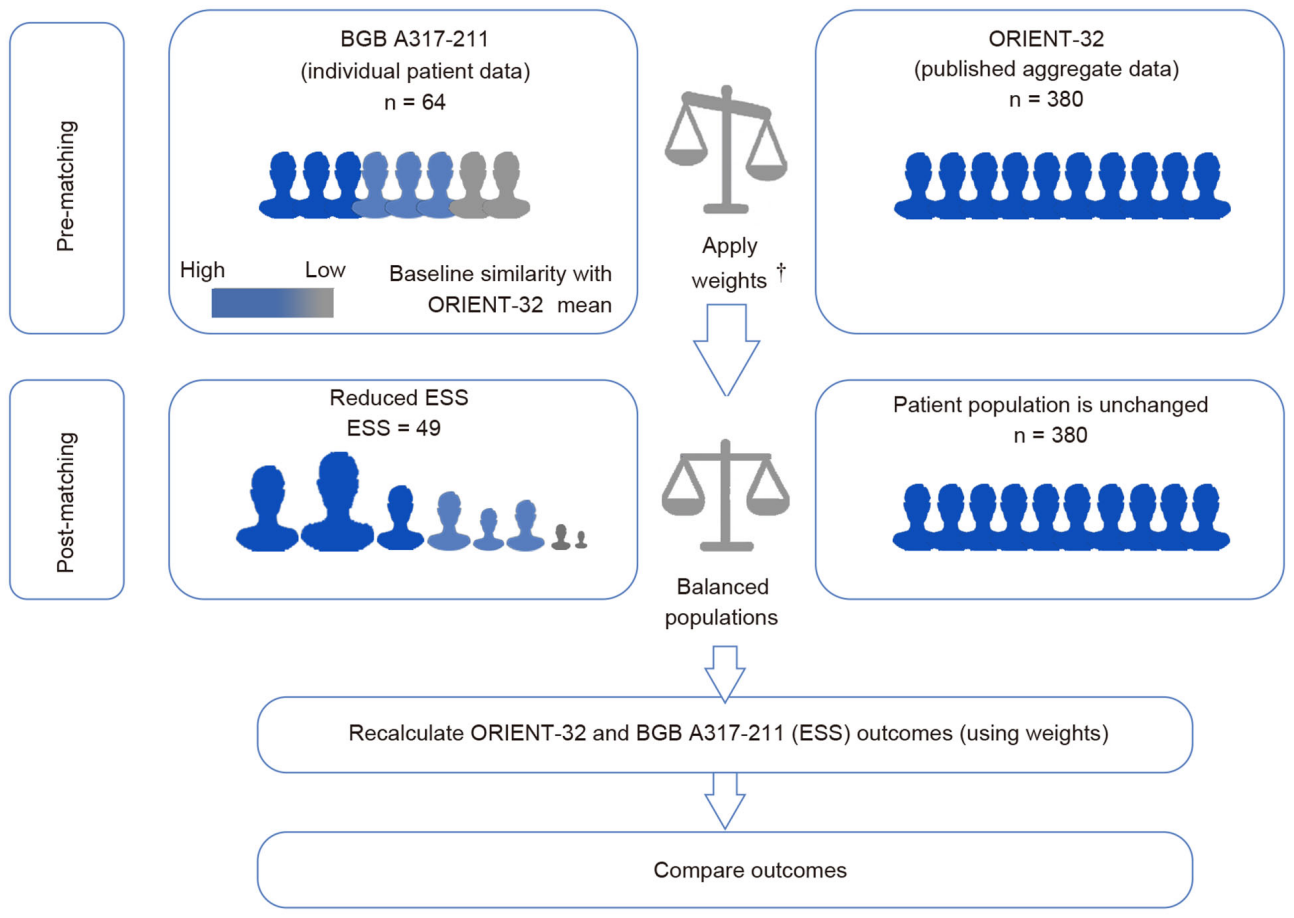
Matching-adjusted indirect comparison methodology infographic. †Weights of individual patients were determined using the method of moments. The choice of matching parameters was identified based on the literature and discussions with clinical experts. Patients were matched for key characteristics known or expected to influence clinical outcomes in individuals with hepatocellular carcinoma.

Unanchored MAICs were conducted to demonstrate the relative efficacy comparison of tislelizumab plus lenvatinib to sintilimab plus bevacizumab biosimilar. The first step when implementing MAIC was to align the patient population of the trials to be compared. Patients across two trials were matched on available potential effect modifiers and prognostic variables, including age, sex, Eastern Cooperative Oncology Group (ECOG) performance status (0 or 1), stage of Barcelona Clinic Liver Cancer (BCLC; B or C), baseline alpha-fetoprotein level (< 400 ng/mL or ≥ 400 ng/mL), and presence of macrovascular invasion and/or extrahepatic metastasis. Baseline characteristics that could be potential modifiers of efficacy outcomes were identified based on the literature (18–21) and discussions with clinical experts. The weight of individual patients was determined using the method of moments, following the published guidelines from the NICE DSU (**Figure 1**).

For binary outcomes (ORR and DCR), a weighted logistic regression model was fitted with a binary treatment indicator for the adjusted patients receiving tislelizumab plus lenvatinib and patients receiving sintilimab plus bevacizumab biosimilar. The odds ratio (OR) was estimated along with 95% CIs. For time-to-event endpoints (PFS and OS), the comparative efficacy of tislelizumab plus lenvatinib versus sintilimab plus bevacizumab biosimilar was estimated as HRs derived from a weighted Cox proportional hazards model with a binary treatment indicator (ie, tislelizumab plus lenvatinib versus sintilimab plus bevacizumab biosimilar). To fit this model under usual circumstances, IPD from both trials would be required. In place of IPD for ORIENT-32, pseudo-IPD for PFS and OS were derived by digitizing published Kaplan-Meier survival curves and using the Guyot et al., 2012 approach (22). In the Cox regression model, BGB-A317-211 IPD values were assigned weights as defined above, while pseudo-IPD values for ORIENT-32 were left unweighted (weights for pseudo-observations were set to 1).

In addition to the MAIC analysis, sensitivity analyses were conducted to demonstrate the stability of the indirect comparison results. The sensitivity analyses consisted of two parts: a leave-one-out sensitivity analysis (each matching covariate was removed sequentially in the population matching for MAIC) and an STC analysis; for the STC analysis, the regression model was fitted between the outcome and variables included in MAIC, then simulated outcomes treated with tislelizumab plus lenvatinib were compared to the outcomes with the ORIENT-32 study sintilimab plus bevacizumab biosimilar group in the comparator’s population. The 95% CI was obtained by bootstrap resampling (stratified bootstrap for DCR, as there are limited number of patients with progressive diseases). All analyses were carried out in R version 4.0.2.

## 3 Results

### 3.1 Matching patient baseline characteristics

Before matching, the baseline characteristics were generally comparable between tislelizumab plus lenvatinib and sintilimab plus bevacizumab biosimilar groups, except that a higher proportion of patients in BGB-A317-211 study had an ECOG performance status of 0 and BCLC stage of B, and a lower proportion with macrovascular invasion and/or extrahepatic metastasis. After matching, the baseline characteristics were balanced between the two groups (**Table 2**). The original sample size of the tislelizumab plus lenvatinib group (N = 62) was reduced by 20.97% after matching (effective sample size = 49).

### 3.2 Efficacy outcomes

After matching, the tislelizumab plus lenvatinib group showed a significantly higher ORR (39.8% vs. 21.0%; OR = 2.56, 95% CI 1.40-4.63; *p* = 0.0027) per RECIST v1.1, compared to the sintilimab plus bevacizumab biosimilar group. A similarly significant higher ORR was also observed based on mRECIST criteria, with an OR of 2.70 (46.5% vs. 24%; 95% CI 1.50-4.84; *p* = 0.0010). Furthermore, DCR was significantly higher in the tislelizumab plus lenvatinib group compared to the sintilimab plus bevacizumab biosimilar group (RECIST v1.1: OR = 3.81, 95% CI 1.62-11.20, *p* = 0.0013; mRECIST: OR = 3.64, 95% CI 1.55-10.71, *p* = 0.0019). Efficacy outcomes comparisons between the two studies are summarized in **Table 3**.

**Table 3:** Summary of the comparison outcomes between the BGB-A317-211 and ORIENT-32 studies.

Outcomes	Tislelizumab plus lenvatinib					Sintilimab plus bevacizumab biosimilar
	Before matching		After matching			
Response, % (95% CI)	N = 62	OR vs. SIN plus BEV (95% CI)	ESS = 49	OR vs. SIN plus BEV (95% CI)	*p*	N = 365 (efficacy evaluable analysis set)
ORR						
RECIST v1.1	38.70 (26.60-51.90)	2.44 (1.38-4.32)	39.80 (26.20-54.80)	2.56 (1.40-4.63)	0.0027	21 (17-25)
mRECIST	46.80 (34.00-59.90)	2.74 (1.57-4.76)	46.50 (32.10-61.30)	2.70 (1.50-4.84)	0.0010	24 (20-29)
DCR						
RECIST v1.1	90.30 (80.10-96.40)	3.57 (1.49-8.54)	90.90 (79.20-97.20)	3.81 (1.62-11.20)	0.0013	72 (67-77)
mRECIST	90.30 (80.10-96.40)	3.41 (1.43-8.17)	90.90 (79.20-97.20)	3.64 (1.55-10.71)	0.0019	73 (68-78)
Survival, median (95% CI), months	N = 62	HR vs. SIN plus BEV (95% CI)	ESS = 49	HR vs. SIN plus BEV (95% CI)	*p*	N = 380 (ITT analysis set)
PFS	8.20 (6.80-NE)	0.59 (0.41-0.85)	9.60 (6.70-NE)	0.56 (0.37-0.84)	0.0054	4.6 (4.1-5.7)
OS	NR	0.38 (0.21-0.70)	NR	0.43 (0.25-0.74)	0.0023	NR

TIS, tislelizumab; LEN, lenvatinib; SIN, sintilimab; BEV, bevacizumab; ORR, objective response rate; DCR, disease control rate; ESS, effective sample size; PFS, progression-free survival; OS, overall survival; RECIST, response evaluation criteria in solid tumors; mRECIST, modified RECIST; NE, not evaluable; NR, not reached; CI, confidence interval; OR, odds ratio; HR, hazard ratio; ITT, intention-to-treat.

**Figure 2:**
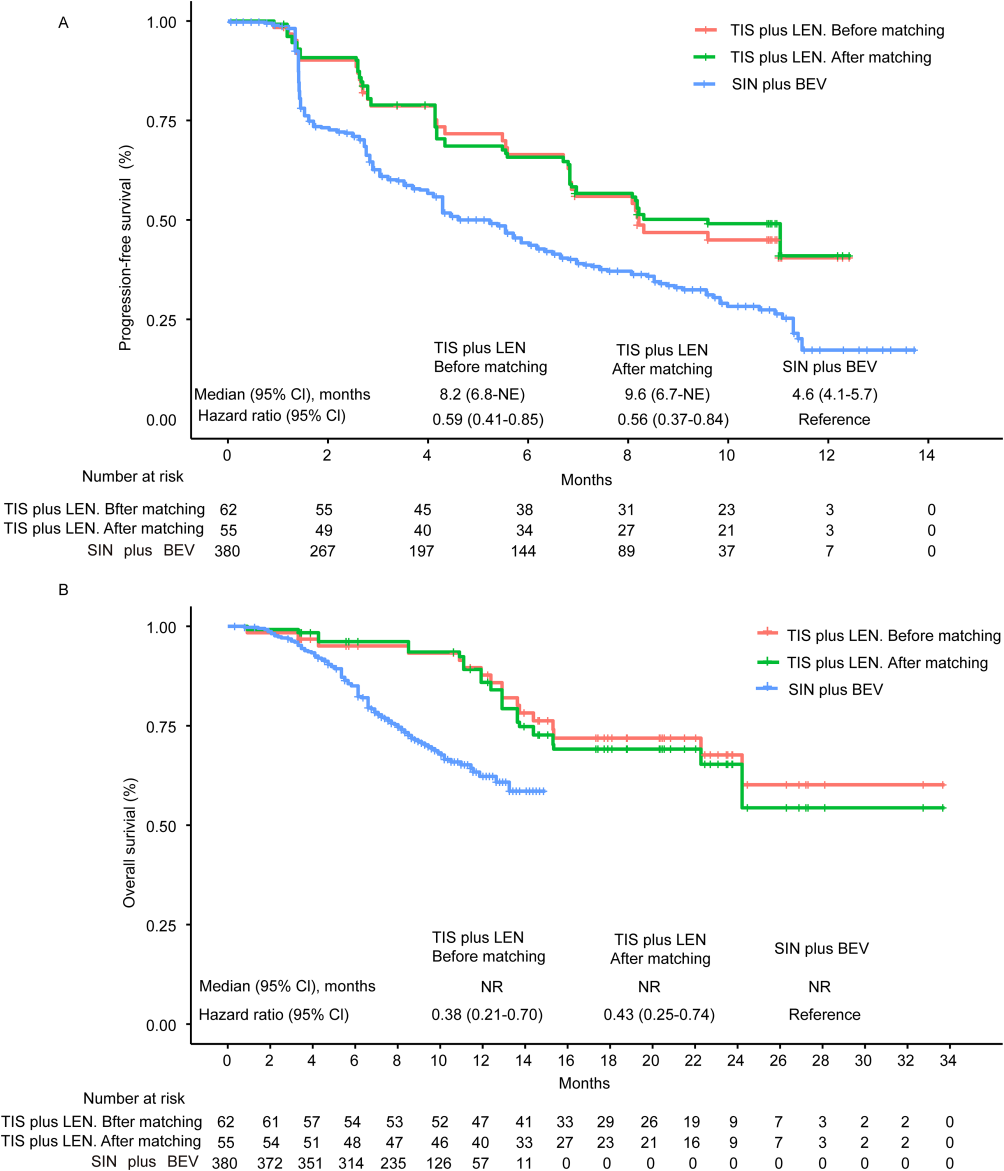
Kaplan-Meier curves of progression-free survival **(A)** and overall survival **(B)** from the MAIC analysis. Kaplan–Meier survival curves (PFS and OS) between TIS plus LEN (before or after matching) and SIN plus BEV showed clear separation and revealed a better survival benefit of TIS plus LEN. NE, not evaluable; NR, not reached; CI, confidence interval; TIS, tislelizumab; LEN, lenvatinib; SIN, sintilimab; BEV, bevacizumab.

The Kaplan-Meier curves of median PFS and OS are illustrated in **Figure 2**. After matching, tislelizumab plus lenvatinib showed a significantly longer PFS than that of the sintilimab plus bevacizumab biosimilar group (9.6 vs 4.6 months, HR = 0.56, 95% CI 0.37-0.84, *p* = 0.0054). Likewise, for median OS, the tislelizumab plus lenvatinib group was associated with a significantly lower risk of death than the sintilimab plus bevacizumab biosimilar group (median not reached [NR] vs. NR, HR = 0.43, 95% CI 0.25-0.74, *p* = 0.0023) after matching.

### 3.3 Sensitivity analysis

**Figure 3:**
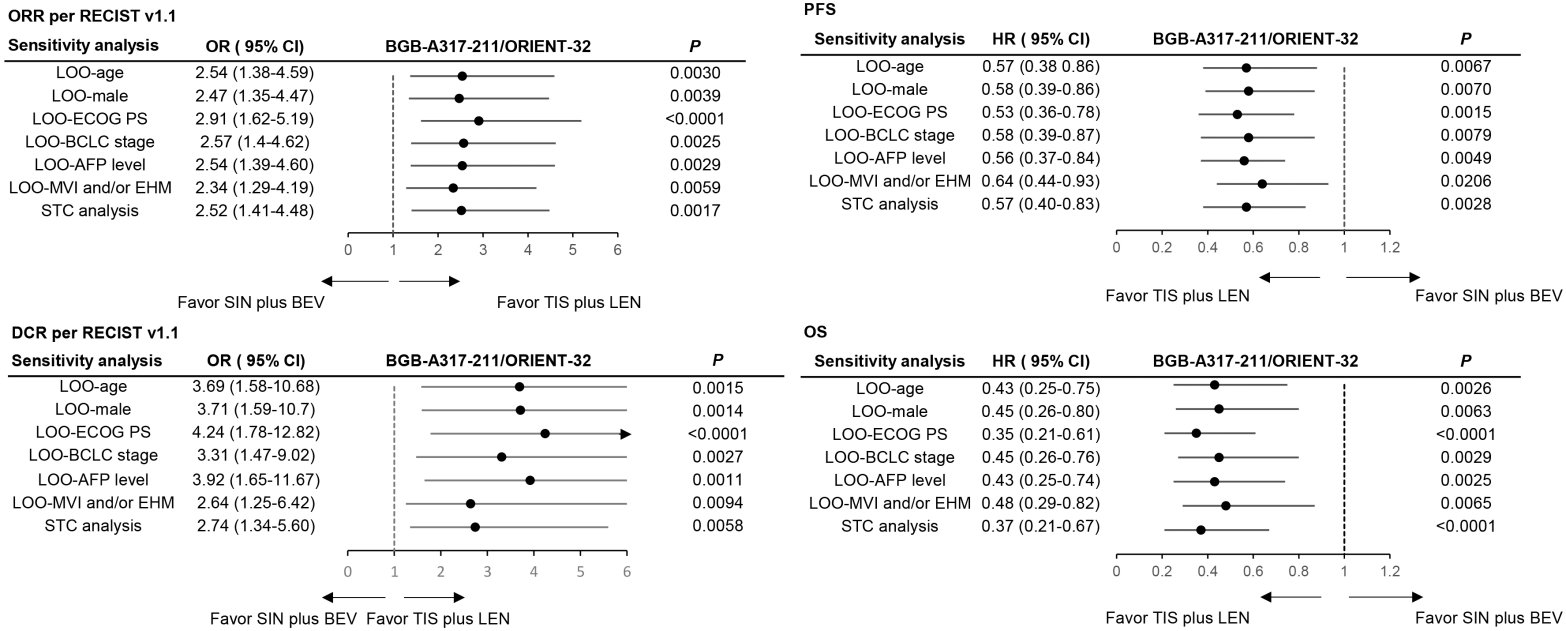
Sensitivity analysis of objective response rate, disease control rate, progression-free survival per RECIST v1.1, and overall survival. Sensitivity analysis results were generally aligned with the significant findings from the MAIC analysis. LOO-age, refers to performing a leave-one-out (LOO) analysis while excluding the “age” variable. LOO-male, excluded the “male” variable. LOO-ECOG PS, excluded the “ECOG PS” variable. LOO-BCLC status, excluded the “BCLC status” variable. LOO-AFP, excluded the “alpha-fetoprotein level” variable. LOO-MVI and/or EHM, excluded the “MVI and/or EHM” variable. STC, simulated treatment comparison. TIS, tislelizumab; LEN, lenvatinib; SIN, sintilimab; BEV, bevacizumab; ECOG PS, Eastern Cooperative Oncology Group Performance Status; BCLC, Barcelona Clinic Liver Cancer stage; AFP, alpha-fetoprotein; MVI, macrovascular invasion; EHM, extrahepatic metastasis. DCR, disease control rate; ORR, objective response rate; PFS, progression-free survival; OS, overall survival; OR, odds ratio; HR, hazard ratio.

Sensitivity analysis results were aligned with the significant findings from the MAIC analysis, indicating the stability of the MAIC results. Forest plots of all sensitivity results are displayed in **Figure 3**; details of ORR and DCR per mRECIST can be found in **Supplementary Figure S1**.

## 4 Discussion

This study used an MAIC approach to compare efficacy outcomes of tislelizumab plus lenvatinib versus sintilimab plus bevacizumab biosimilar for uHCC patients in the first-line treatment setting. The MAIC results showed improvement both in treatment response (ORR: OR = 2.56, 95% CI 1.40-4.63, *p* = 0.0027; DCR: OR = 3.81, 95% CI 1.62-11.2, *p* = 0.0013), and survival benefits (PFS: HR = 0.56, 95% CI 0.37-0.84, *p* = 0.0054; OS: HR = 0.43, 95% CI 0.25-0.74, *p* = 0.0023) with tislelizumab plus lenvatinib. The results were validated by sensitivity analysis, showing the robustness of the study.

Understanding the comparative effectiveness of tislelizumab plus lenvatinib and sintilimab plus bevacizumab biosimilar is of interest to help inform clinical decisions and maximize patient benefit. We conducted an MAIC analysis to indirectly compare these two treatments due to the absence of head-to-head randomized trials. The findings of the study were reliable. Firstly, the MAIC approach utilized in this study is deemed a robust statistical approach, as it can adjust for confounders caused by cross-trial differences or effect modifiers, maximizing the reduction of bias in the indirect treatment comparison results (23, 24). Notably, though STC is believed to potentially have less bias when used in unanchored scenarios, the implementation of STC can be limited by a relatively small sample size compared to the number of covariates adjusted in the outcome model, which can lead to unstable parameter estimations. Consequently, MAIC is a more suitable approach for population adjustment in this analysis, despite the potential for increased variation. Secondly, we estimated weights for the IPD from BGB-A317-211 for alignment with weighted baseline characteristics of the comparator population from ORIENT-32 by strictly following the guidelines issued by the NICE DSU (19). Thirdly, sensitivity analyses were performed to test the stability of MAIC results, and they supported the robustness of MAIC findings. The sensitivity analyses consisted of two portions. On the one hand, the leave-one-out analysis indicated that the findings from the MAIC analysis were robust and not overly dependent on any single covariate. On the other hand, STC adjusted the population through an outcome regression model. The results supported the MAIC results. Together, the MAIC results are robust to suggest that tislelizumab plus lenvatinib may provide better clinical benefit than sintilimab plus bevacizumab biosimilar as a first-line treatment for Chinese patients with uHCC.

In China, about 70%-80% of HCC patients are diagnosed at advanced stages and are ineligible for hepatic resection (25, 26). Thus, selecting the optimal systemic treatment regimen that offers better survival benefits for Chinese patients with uHCC is an urgent need to be addressed. The difference in effectiveness between tislelizumab plus lenvatinib and sintilimab plus bevacizumab biosimilar can only be conclusively determined through a direct head-to-head prospective clinical trial. However, this MAIC may inform treatment decisions in Asian patients with HCC, in the absence of head-to-head clinical trial data. This efficacy benefit observed in our study was supported by contemporary phase 3 studies of PD-1 inhibitors plus TKIs in CARES 310 and the Asian population subgroup analysis from LEAP 002 (8, 27). The favorable outcomes in the Asian population may be attributed to the majority of HCC cases in Asia being associated with hepatitis B virus (HBV) infection. Immune checkpoint inhibitor therapies could highly benefit HBV-related HCC patients by reactivating the exhausted immune cells and blocking the immune checkpoint molecules in the tumor microenvironment (28, 29). Besides this, mechanistic studies have provided a potential biological rationale for the enhanced efficacy of tislelizumab plus lenvatinib (30). The pathogenesis of HBV-related HCC is complex and involves multiple molecular players and intertwined signaling pathways, such as virus-host genome integration, sustained inflammation due to the host’s immune reaction, and cellular signal transduction pathways altered by the HBV-encoded oncogene X protein (HBx) (31, 32); HBx plays a significant role in liver cancer by modulating several cancer-related signaling pathways, such as mitogen-activated protein kinase (MAPK), rat sarcoma virus, rapidly accelerated fibrosarcoma, focal adhesion kinase, and kinase C signaling cascades (33). These findings collectively indicate that multi-target drugs with their inherent ability to inhibit multiple pathways may be superior to single-target inhibitors (34). Lenvatinib is a multi-targeted TKI inhibiting vascular endothelial growth factor receptors 1-3, fibroblast growth factor receptor 1-4, C-KIT, RET protooncogene, and platelet-derived growth factor receptor α (35, 36). Aside from blocking VEGF pathways, lenvatinib disrupts several other key receptors involved in cancerous signaling, particularly the MAPK and phosphatidylinositol-3-kinase/AKT/mammalian target of rapamycin (mTOR) (35–39). However, as the analysis is based on an unanchored MAIC rather than a head-to-head randomized trial, the results should be interpreted as indicative rather than definitive.

The safety results from the two studies indicated that treatment-related adverse events occurred at rates of 95.3% (any grade), 28.1% (grade 3-4), and 0 (grade 5) for tislelizumab plus lenvatinib, and 89% (any grade), 34% (grade 3-4), and 2% (grade 5) for sintilimab plus bevacizumab biosimilar (11, 16). A safety comparison is limited by differing median treatment durations between BGB-A317-211 (tislelizumab: 11.0 months; lenvatinib: 11.1 months) and ORIENT-32 (sintilimab: 7.0 months; bevacizumab biosimilar: 6.6 months). These discrepancies in treatment duration may lead to differences in drug exposures, and adjustments with drug exposure of each individual patient are necessary to fairly compare the safety of these two treatment regimens. Given that this is an indirect head-to-head comparison, the results should be interpreted with caution. Further validations on the clinical efficacy and safety of tislelizumab plus lenvatinib and sintilimab plus bevacizumab biosimilar in the first-line treatment of uHCC are warranted.

This study also had some limitations. While MAIC procedures can reduce the impact of potentially effect-modifying baseline characteristics for reported covariates, they were not able to adjust for between-trial differences, such as in study design with the BGB-A317-211 as a phase 2 single-arm study with a small sample size and lack of blinding, even though the BGB-A317-211 study employed an IRC, which may mitigate potential bias from investigator assessments and enhance the reliability of the findings. Between-trial differences in the tumor response assessment schedules could also not be fully adjusted in the analyses. The BGB-A317-211 study had 6-weekly disease assessments in the first year of treatment, followed by assessments every 9 weeks thereafter, while the ORIENT-32 study had 6-weekly assessments for 2 years and then every 12 weeks. This may yield biased longer PFS or OS in the latter study. Additional variables, such as prior local-regional therapy, were also not able to be adjusted. Despite both studies reporting the prior local regional therapy (e.g., TACE, ablation, radiotherapy), the heterogeneity in treatment type, and intensity lead to practically infeasible to harmonize or match this variable across studies. Such differences are unavoidable features of some indirect treatment comparisons (40, 41) and network meta-analyses (42, 43), but are relevant factors to consider when interpreting their results.

There are additional limitations in this MAIC analysis worthy of consideration. Firstly, in unanchored MAIC for survival outcomes where IPD were unavailable for comparator trials, pseudo IPD was reconstructed through digitization of published Kaplan–Meier curves; however, true patient-level data cannot be perfectly replicated. This also serves as another unavoidable feature of indirect treatment comparisons (40, 41). In this study, to minimize such bias and ensure the reliability of the reconstructed data, we compared key reconstructed survival metrics against the reported summary statistics. Specifically, we confirmed that the median progression-free survival derived from the pseudo-IPD (4.6 months [95% CI: 4.3–5.9]) closely matched the reported value in ORIENT-32 (4.6 months [95% CI: 4.1–5.7]). This validation approach supports the accuracy of the reconstruction and enhances the robustness of our findings. Secondly, both studies had limited follow-up durations, and neither reached a mature OS, which requires caution when interpreting the OS results. Thirdly, subsequent treatments may influence overall survival outcomes, given the different study periods of the BGB-A317-211 trial (conducted from September 4, 2020 to January 7, 2022) and the ORIENT-32 trial (conducted from February 11, 2019 to January 15, 2020), along with advancements in systemic therapies in recent years. The inability to account for differences in subsequent therapy may impact the assessment of survival outcomes. This limitation is consistent with previous publications that have also recognized the impact of subsequent therapy due to variability in clinical practice and limited standardized reporting across trials (44, 45). Fourthly, the reduced effective sample size resulting from MAIC matching and adjustments decreases the statistical power of subsequent analyses, representing a challenge commonly encountered in similar studies (46), despite that sensitivity analysis using bootstrap resampling confirmed the robustness of the treatment effect estimates. Taken together, for these reasons, the results of an MAIC cannot replace evidence from a randomized controlled trial.

In conclusion, the present analysis suggested that after adjusting for relevant prognostic factors and treatment effect modifiers, tislelizumab plus lenvatinib showed favorable clinical benefit compared to sintilimab plus bevacizumab biosimilar in uHCC first-line treatment. Sensitivity analyses also supported the MAIC analysis results. This study provided a reference to assist physicians in choosing first-line treatment regimens for Chinese patients with uHCC. Further randomized controlled trials are warranted to validate the findings of this analysis.

## Data Availability

The original contributions presented in the study are included in the article/**Supplementary Material**. Further inquiries can be directed to the corresponding authors.
