# Supplementary material for: Matching-adjusted indirect comparison of tislelizumab plus lenvatinib versus sintilimab plus bevacizumab biosimilar as first-line treatment for unresectable hepatocellular carcinoma
Source: Front Immunol. 2025 Jun 23;16:1594935. doi: 10.3389/fimmu.2025.1594935 (PMC12230042; doi:10.3389/fimmu.2025.1594935)
Supplement: Supplementary file 1 [file DataSheet1.docx]

**Supplementary Materials**


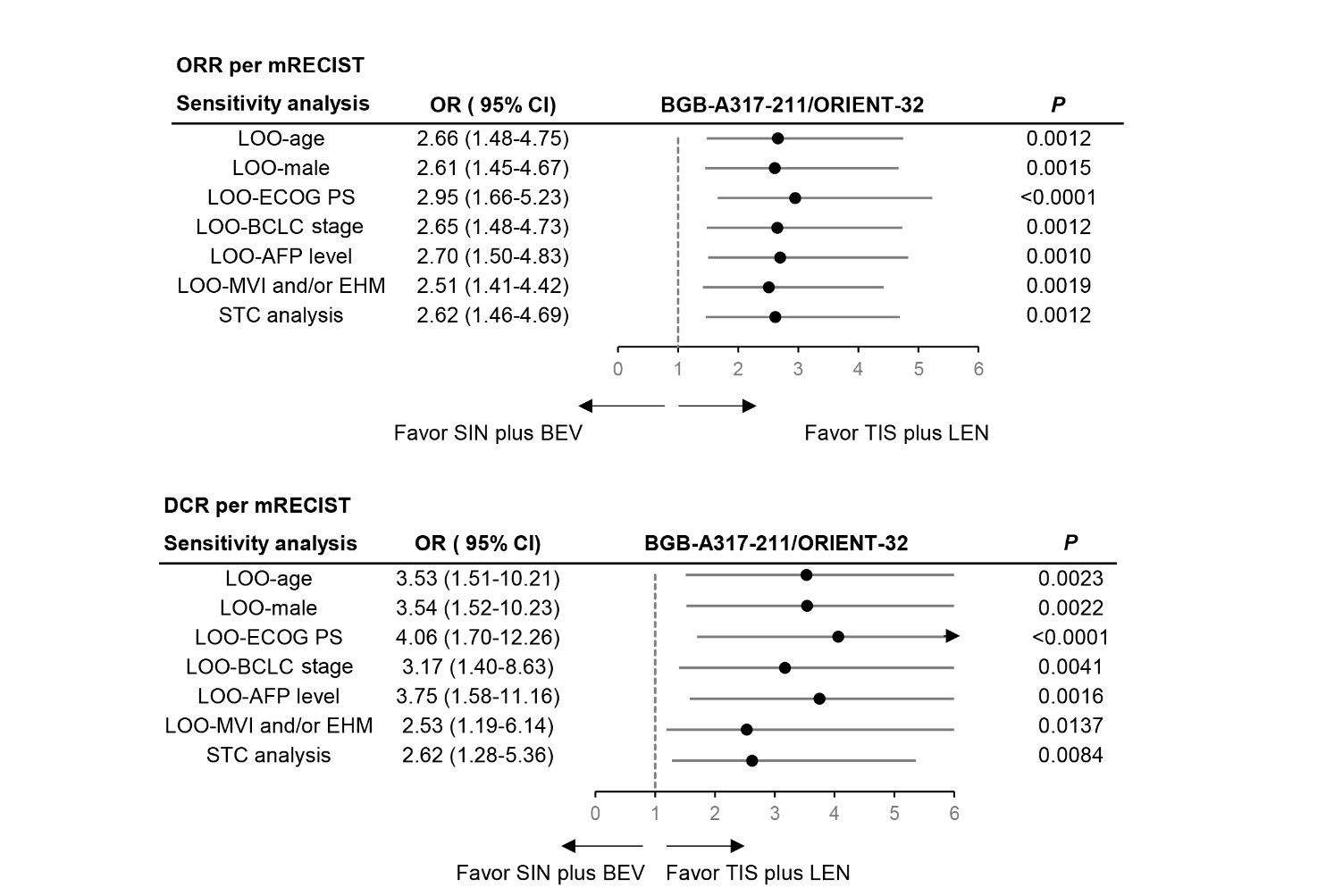


**Figure S1**

**Sensitivity analysis of objective response rate, disease control rate per modified RECIST.** Sensitivity analysis results were generally aligned with the significant findings from the MAIC analysis. LOO-age, refers to performing a leave-one-out (LOO) analysis while excluding the “age” variable. LOO-male, excluded the “male” variable. LOO-ECOG PS, excluded the “ECOG PS” variable. LOO-BCLC status, excluded the “BCLC status” variable. LOO-AFP, excluded the “alpha-fetoprotein level” variable. LOO-MVI and/or EHM, excluded the “MVI and/or EHM” variable. STC, simulated treatment comparison. TIS, tislelizumab; LEN, lenvatinib; SIN, sintilimab; BEV, bevacizumab; ECOG PS, Eastern Cooperative Oncology Group Performance Status; BCLC, Barcelona Clinic Liver Cancer stage; AFP, alpha-fetoprotein; MVI, macrovascular invasion; EHM, extrahepatic metastasis. DCR, disease control rate; ORR, objective response rate; OR, odds ratio.
